# Diversity, Virulence, and Antimicrobial Resistance in Isolates From the Newly Emerging *Klebsiella pneumoniae* ST101 Lineage

**DOI:** 10.3389/fmicb.2019.00542

**Published:** 2019-04-02

**Authors:** Chandler C. Roe, Adam J. Vazquez, Eliana Pia Esposito, Raffaele Zarrilli, Jason W. Sahl

**Affiliations:** ^1^Pathogen and Microbiome Institute, Northern Arizona University, Flagstaff, AZ, United States; ^2^Department of Public Health, University of Naples Federico II, Naples, Italy

**Keywords:** *Klebsiella*, phylogenetics, antimicrobial resistance, ST101, genomics

## Abstract

The global dissemination of *Klebsiella pneumoniae* and *Klebsiella pneumoniae* carbapenemase (KPC) has been largely attributed to a few high-risk sequence types (STs) (ST258, ST11, ST512) associated with human disease. ST101 is an emerging clone that has been identified in different parts of the world with the potential to become a global, persistent public health threat. Recent research suggests the ST101 lineage is associated with an 11% increase in mortality rate in comparison to non-ST101 infections. In this study, we generated a high-quality, near-finished genome assembly of a multidrug-resistant (MDR) isolate from Italy (isolate 4743) that is a single locus variant of ST101 (ST1685). We demonstrate that the 4743 genome contains virulence features such as an integrative conjugative element carrying the yersiniabactin siderophore (ICEKp3), the mannose-resistant *Klebsiella*-like (type III) fimbriae cluster (mrkABCDFHIJ), the ferric uptake system (kfuABC), the yersiniabactin receptor gene *fyuA*, a capsular K type K17, and an O antigen type of O1. *K. pneumoniae* 4743 carries the *bla*KPC-2 carbapenemase gene along with genes conferring resistance to aminoglycosides, beta-lactams, fluoroquinolones, fosfomycin, macrolides, lincosamides, and streptogramin B. A comparative genomics analysis of 44 ST101 genomes as well as newly sequenced isolate 4743 identified variable antimicrobial resistance (AMR) resistance profiles and incompatibility plasmid types, but similar virulence factor profiles. Using Bayesian methodologies, we estimate the common ancestor for the ST101 lineage emerged in 1990 (95% HPD: 1965 to 2007) and isolates within the lineage acquired *bla*_KPC_ after the divergence from its parental clonal group and dissemination. The identification of virulence factors and antibiotic resistance genes acquired by this newly emerging clone provides insight into the reported increased mortality rates and highlights its potential success as a persistent nosocomial pathogen. With a combination of both colistin resistance, carbapenem resistance, and several known virulence factors, the ST101 genetic repertoire may be a “perfect storm” allowing for a newly emerging, high-risk, extensively antibiotic resistant clone. This high-risk clone appears adept at acquiring resistance and may perpetuate the dissemination of extensive antimicrobial resistance. Greater focus on the acquisition of virulence factors and antibiotic resistance genes is crucial for understanding the spread of antibiotic resistance.

## Introduction

The Gram-negative bacterium, *Klebsiella pneumoniae*, is abundantly distributed in the environment and has traditionally been considered an opportunistic pathogen associated with hospital-acquired and community-acquired infections (Holt et al., 2015). Also, the rapid and global dissemination of multidrug-resistant (MDR) and extensively drug resistant (XDR or pan-resistant) *Klebsiella pneumoniae* was recently recognized by the CDC as an urgent public health threat requiring immediate and aggressive action (CDC, Antibiotic resistance threats in the U.S., 2013). Currently, carbapenem and broad-spectrum antibiotics are considered last resort treatment options, but the increasing incidence of extended-spectrum beta-lactamase (ESBL) and carbapenem resistant (CRE) isolates coupled with their global distribution highlights the potential for rapid dissemination of mobile MDR genes to other highly virulent, nosocomial pathogens (Ventola, 2015). *K. pneumoniae* contains significant genome variability and large accessory genomes, which includes virulence functions associated with invasive disease in humans and antimicrobial genes associated with hospital-acquired infections (Bialek-Davenet et al., 2014a; Holt et al., 2015; Cerqueira et al., 2017). Recent genomic epidemiology studies of *K. pneumoniae* isolates identified two types of high-risk clonal groups among bacterial populations: hyper-virulent clonal complexes (CCs) responsible for community-acquired invasive infections and multidrug-resistant CCs responsible for health-care associated infections (Struve et al., 2015; Cerqueira et al., 2017). The existence of hyper-virulent clones is a key feature of *K. pneumoniae* and typically show increased pathogenesis not commonly associated with antimicrobial resistance (Struve et al., 2015).

*Klebsiella pneumoniae* isolates responsible for health-care associated infections are usually CRE due to production of either class B metallo-β-lactamases (MBLs) (IMP,VIM,NDM) or class A (KPC) or class D (OXA-48) serine carbapenemases (Pitout et al., 2015; Grundmann et al., 2017; Logan and Weinstein, 2017). Multidrug-resistant (MDR) *K. pneumoniae* isolates have primarily been assigned to CC258 (Gaiarsa et al., 2015) and to additional emerging genotypes ST11, ST15 and ST101 (Bialek-Davenet et al., 2014a; Bowers et al., 2016; Conte et al., 2016; Cerqueira et al., 2017; Moradigaravand et al., 2017). *K. pneumoniae* isolates associated with hospital-acquired infections have acquired antimicrobial resistance genes and are usually devoid of virulence genes (Bialek-Davenet et al., 2014a; Cerqueira et al., 2017). However, the acquisition of yersiniabactin (an iron sequestering system crucial for disease establishment) has been observed in many isolates of the epidemic KPC-producing CC258 (Holt et al., 2015). High-risk clones have a highly flexible accessory genome and are adept at both acquiring resistance as well as switching resistance profiles (Woodford et al., 2011). Their global success as a nosocomial pathogen can be correlated with excessive genome plasticity (Conlan et al., 2016). Understanding high-risk clones’ ability to adapt and survive in a hospital environment is vital to assuage further spread of antibiotic resistance. While hyper-virulent and drug-resistant *K. pneumoniae* populations remain mostly non-overlapping, combinations of these groups (isolates within CC23) have been described (Bialek-Davenet et al., 2014a). The risk of combination of these two high-risk genetic profiles highlights the importance of understanding the spread of genomic regions from the *K. pneumoniae* accessory genome.

Among the newly emerging genotypes, *K. pneumoniae* isolates belonging to ST101 are associated with hospital-acquired infections and epidemics worldwide (Giani et al., 2013; Skálová et al., 2016; Gonçalves et al., 2017). *K. pneumoniae* isolates assigned to ST101 are CRE because of the production of KPC-2 (Oteo et al., 2016) or OXA-48 (Skálová et al., 2016; Avgoulea et al., 2018). Also, the emergence of colistin resistance has been observed in KPC-2 producing (Del Franco et al., 2015) and OXA-48 producing (Giani et al., 2013; Del Franco et al., 2015; Papagiannitsis et al., 2016) *K. pneumoniae* ST101 isolates. The focus of this study was to characterize the phylogenetic and genomic diversity within the emerging nosocomial high-risk sequence type ST101. MDR *K. pneumoniae* isolates typically belong to specific high-risk sequence types (ST11, ST258, ST512) (Bialek-Davenet et al., 2014a), with extensive comparative genomics research investigating the genomic backbone of these clones, however, few studies have investigated the global genomic diversity of the newly emerging and clinically relevant ST101 clone (Gonçalves et al., 2017; Moradigaravand et al., 2017; Avgoulea et al., 2018). In this study, we investigated the lineage relatedness, resistance determinants, plasmid profiles, core and accessory genome content, and the evolutionary rate of the ST101 sequence type through whole-genome sequencing. We propose a timeline for emergence as well as provide a better understanding of the evolution and composition of an increasingly successful MDR global lineage that may aid in the identification of newly emerging novel *K. pneumoniae* lineages and help understand the proliferation of extensively antibiotic resistance pathogens.

## Materials and Methods

### Isolate Information of an MDR Strain From Italy

*Klebsiella pneumoniae* 4743 strain was isolated from rectal swab of a patient in the intensive care unit ward of public hospital of USL Valle D’Aosta, Aosta, Italy on November 24th 2013. To compare this isolate in a phylogenetic context, a global collection of 1,723 publicly available *K. pneumoniae* isolates were downloaded and analyzed (Supplementary Table S1).

### Antimicrobial Resistance Profiling

Antimicrobial susceptibilities were performed for 4743 using the Vitek 2 system and the AST-GN card (bioMérieux, Marcy l’Étoile, France). Values were interpreted according to breakpoint table for interpretation of MIC values and zone diameters (European Committee on Antimicrobial Susceptibility Testing, 2016). Colistin susceptibility assay was performed according to recommendations of joint CLSI-EUCAST guidelines: http://www.eucast.org/fileadmin/src/media/PDFs/EUCAST_files/General_documents/Recommendations_for_MIC_determination_of_colistin_March_2016.pdf.

### DNA Extraction, Sequencing, and Assembly

For Illumina MiSeq sequencing of strain 4743, genomic DNA was extracted with the GenElute DNA extraction kit (Sigma-Aldrich, Milan, Italy). A sequence library was generated for paired-end sequencing using previously described methods (Stone et al., 2016) and sequenced to an average depth of 50*x*. For PacBio sequencing, genomic DNA of sample 4743 was extracted using a DNeasy Blood and Tissue Kit according to the manufacturer’s instructions (Qiagen, Milan, Italy). Approximately 10 μg of DNA was fragmented to 10–20 kbp using the G-tube apparatus (Covaris) following the manufacturer’s recommendations. A PacBio Sequencing library was constructed using the SMRTbell^TM^ Template Prep Kit 1.0 and by following the PacBio 20 kb library protocol; the library molecules, after adapter ligation and damage repair, were size selected for 15 kb and larger using Blue Pippin instrument (Sage Sciences) by following the manufacturer’s instructions. The final library was processed for sequencing by using PacBio MagBead kit v2 with the P6/C4 chemistry and following PacBio protocols. Sequencing was performed on PacBio RSII instrument in one SMRT cell (v3) for 6 h.

The 4743 genome was assembled using Unicycler v0.4.7 (Wick et al., 2017), resulting in seven contigs. The genome was polished by running six rounds of Pilon (Walker et al., 2014) and was then processed with Circlator (Hunt et al., 2015). Assembly statistics are shown in Supplementary Table S2. The assembly and raw reads were deposited in NCBI under BioProject PRJNA477005. Additionally, genome annotation was performed using PROKKA v1.13 (Seemann, 2014).

### *In silico* MLST Typing

Multi-locus sequence typing (MLST) analysis was performed on all genome assemblies using an *in silico* MLST script^1^ which implemented the Institut Pasteur’s MLST scheme as previously described (Diancourt et al., 2005).

### *In silico* K Typing and O Typing

Polysaccharide capsule (K typing) and lipopolysaccharide O antigen typing was performed using the Kaptive tool (Wyres et al., 2016). Briefly, the source code for the command-line version of Kaptive was downloaded from the GitHub repository^2^. Both K and O typing of 45 ST101 isolates was accomplished running default settings within the Kaptive.py script.

### Core Genome SNP Phylogenies

All external *K. pneumoniae* genomes (*n* = 3,352) were downloaded from GenBank in February 2018. Genomes were filtered from the dataset if they contained: (1) greater than 10 ambiguous nucleotides; (2) an anomalous number of contigs (>462); (3) an anomalous genome assembly size (<5,195,738, >5,992,749) and; (4) an anomalous pairwise MASH distance (>0.017) (Ondov et al., 2016); this filtering resulted in a final dataset of 1,504 genomes. All genome assemblies were aligned against the completed reference ST101 genome GCA_001902435 using NUCmer (Delcher et al., 2002) and SNPs were identified as part of the NASP pipeline (Sahl et al., 2016). A global maximum likelihood phylogeny was generated using the TVM+ASC+G4 nucleotide substitution model in IQTREE v1.6.1 (Nguyen et al., 2015) with 1,000 bootstrap replicates. Trees were visualized in FigTree^3^. The Retention Index (Farris, 1989) was calculated with Phangorn (Schliep, 2011).

For the ST101 clade analysis, assembled genomes were aligned to sample Kp_Goe_33208 (GCA_001902435) using the previously described methods above. A ST101-only maximum likelihood phylogeny was inferred using the K3P+ASC substitution model with 1,000 bootstrap replicates in IQTREE. Phylogenetic trees were visualized in FigTree. The Retention Index was calculated with Phangorn. Additionally, the presence of recombination within this dataset was determined using the PHI statistic from the package PhiPack (Bruen et al., 2006).

### LS-BSR Analysis

The Large-Scale Blast Score Ratio (LS-BSR) pipeline (Sahl et al., 2014) was used to identify differential coding region conservation within the ST101 clade. This pipeline predicted coding regions (CDSs) using the program Prodigal v2.60 (Hyatt et al., 2010) and clustered the putative CDS regions with 90% identity using VSEARCH v1.11.1 (Rognes et al., 2016). The resulting regions were aligned against themselves using the program BLAT v35×1 (Kent, 2002) to generate a reference bit score. Genomic regions were then aligned back to every sample in the ST101 dataset using BLAT to generate a query bit score. The query bit score was divided by the reference bit score for each region to obtain the BLAST Score Ratio (BSR) (Rasko et al., 2005). The core CDSs were identified as genomic regions that had a BSR value of > 0.80 across all genomes. Unique genomic regions were identified based on a BSR value < 0.40 in all but a single genome.

### Genes Associated With Virulence, Heavy Metal Resistance, and Drug Resistance

Genes associated with virulence (120), colibactins (91), yersiniabactins (165), heavy metal resistance (203), efflux pumps and regulators (489), and non-scheme genes (278) were downloaded from BIGSdb-Kp database (Bialek-Davenet et al., 2014b). LS-BSR was run on the ST101-only lineage with the genes parameter flagged using the virulence, heavy metal resistance, and efflux pump databases as input. LS-BSR was run using nucleotides and the BLAT alignment option. A BSR value > 0.95 was considered present.

### QRDR Mutations

Quinolone resistance determining regions (QRDRs) within the ST101 genomes were inspected for mutations conferring increased fitness within gyrase (*gyrA* and *gyrB*) and topoisomerase IV (*parC* and *parE)* genes. Reference genes for *parE (*AFQ64062.1), *parC* (AFQ64068.1), *gyrA* (AFQ64815.1), and *gyrB* (AFQ63426.1) were downloaded from NCBI. Blastx from blast+ v2.2.29 (Camacho et al., 2009) was used to identify the composition of each gene in the ST101 genomes. Genes were extracted from each genome and alignments were performed using ClustalW (Thompson et al., 1994) in MEGA7 (Kumar et al., 2016).

### Plasmid Analysis

The *Enterobacteriaceae* plasmid database from PlasmidFinder (Carattoli et al., 2014) was used to identify plasmid types within the ST101 clade. The PlasmidFinder database consists of representative genes of varying incompatibility groups. Here we screened 122 typing genes from PlasmidFinder against the ST101 genomes using LS-BSR/BLAT. BSRs were generated and visualized in Rstudio using the shinyheatmap program (Khomtchouk et al., 2017). Typing genes with a BSR value > 0.95 were considered to be present.

### Antibiotic Resistance Gene Screening

The distribution of genes associated with virulence and antimicrobial resistance was identified for all ST101 genomes. Acquired genes associated with antimicrobial resistance were identified using the ResFinder database (Kleinheinz et al., 2014) with LS-BSR. The ResFinder database included genes that may confer resistance to the following antibiotics: aminoglycosides, beta-lactams, colistin, fluoroquinolone, fosfomycin, fusidic acid, glycopeptide, macrolide, lincosamide, and streptogramin B (MLS), nitroimidazole, oxazolidinone, phenicol, rifampicin, sulphonamide, tetracycline, and trimethoprim. Mutations within the *mgr*B regulator gene that are known to confer resistance to colistin were also investigated (Esposito et al., 2018). Briefly, the program BLAT was used to identify the *mgrB* gene within the ST101 samples and the sequence was extracted from each alignment. The *mgrB* gene sequences were aligned using MUSCLE (Edgar, 2004) in MEGA7 (Kumar et al., 2016) and subsequently translated into amino acids. The amino acid sequences were visually inspected for premature stop codons. Additionally, genes associated with virulence were obtained from the BIGSdb-KP database and screened for within the ST101 genomes using LS-BSR. A heatmap representing presence/absence of antimicrobial resistance genes was produced using shinyheatmap in *R*.

### Population Structure

In order to assess the genomic relationships within the ST101 lineage, we applied Plink v1.07 (Purcell et al., 2007) and fastStructure (Raj et al., 2014), a Bayesian model-based clustering algorithm; the Plink output was used as input for fastStructure. Briefly, SNP positions with more than two allele states were removed from the dataset. Plink was implemented using default parameters. As the *K* parameter represents populations in fastStructure, we implemented *K = 2* through *K* = 9 with default parameters. An optimal *K* value was chosen based on model complexity that maximizes marginal likelihood as well as optimum model components used to explain the structure.

### Beast Timing Analysis

BEAST analysis included 36 of the 45 ST101 genomes (Supplementary Table S3). Strains were only included in this analysis if the collection date was listed under the biosample data on NCBI. A core SNP matrix was generated by aligning raw reads to sample 4743 using the NASP pipeline. The presence of recombination within a dataset can confound molecular clock analyses and was therefore identified and removed using ClonalframeML running default parameters (Didelot and Wilson, 2015). A regression analysis implementing root-to-tip genetic distance as a function of the sample collection year was conducted using the software package TempEst version 1.5.1 (Rambaut et al., 2016). A measure of clocklike behavior was assessed using the determination coefficient R^2^ and the rooted ST101-only phylogeny. Additionally, a 10,000 date-randomization permutation of sampling collection dates was performed in an effort to compare our regression coefficient to that observed by random chance (Murray et al., 2016).

## Results

### 4743 Strain Details

*Klebsiella pneumoniae* 4743 was isolated from a rectal swab of a patient admitted to Intensive Care Unit of USL Valle D’Aosta, Aosta, Italy on November 24th 2013. *K. pneumoniae* 4743 isolate was representative of an epidemic of KPC-2 producing *K. pneumoniae* CC101 occurring from November 2013 to August 2014 (Del Franco et al., 2015). *K. pneumoniae* 4743 isolate was able to transfer resistance to carbapenems and ESBL activity along with conjugative plasmids carrying *bla*_KPC–2_ and *bla*_CTX–M–group1_ genes, respectively (Del Franco et al., 2015).

### 4743 Genome Assembly

The genome assembly using PacBio reads combined with Illumina MiSeq reads produced a total of 7 contigs, 6 of which are plasmids and 1 of which is chromosomal; 4 of the plasmids were determined to be circular. The total size of sample 4743 is 5,857,478 bases. PHASTER identified four intact phage within the large chromosome contig, and two more complete phage in two of the plasmid contigs (4743 plasmid unnamed 3 and 4743 plasmid unnamed 6).

### Antimicrobial Resistance Profiling

Antimicrobial susceptibilities were performed for sample 4743 using the Vitek 2 system and the AST-GN card. *Klebsiella pneumoniae* 4743 isolate was resistant to imipenem, meropenem, ertapenem, beta-lactam/beta-lactamase inhibitor combinations (clavulanic acid/amoxicillin, piperacillin/tazobactam), third and fourth generation cephems, ciprofloxacin, but susceptible to trimethoprim-sulfamethoxazole and colistin (Supplementary Table S4).

### External Genomes

External genome assemblies from *K. pneumoniae* were downloaded from the assembly database in GenBank and filtered based on several quality criteria, resulting in 1,504 genome assemblies. *in silico* MLST typing was performed on all assemblies using the previously described Pasteur system (Diancourt et al., 2005). Of all *K. pneumoniae* isolates analyzed, 41 were assigned to ST101, three were assigned to ST2017, and one sample (4743) was assigned to ST1685. Both ST2017 and ST1685 are single locus variants (locus *rpoB*) of ST101 and were included in subsequent analyses.

### Phylogenetics and Comparative Genomics

**FIGURE 1:**
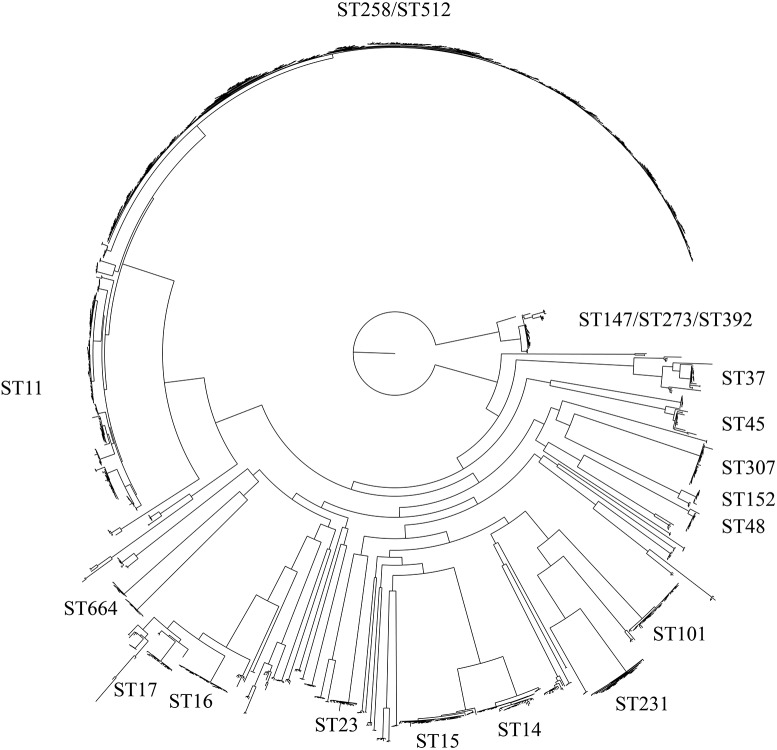
Maximum-likelihood phylogeny of the *Klebsiella pneumoniae* species. This tree includes 1,724 genomes that collectively cover 38.59% of the GCA 001902435 reference genome and is based on 118,011 total SNPs. Within this species, several large clades exist and are labeled according to their MLST type.

WGS of one isolate (4743) was performed and compared to whole genome sequences of 44 *K. pneumoniae* isolates assigned to the ST101 lineage available in GenBank and 1,504 non-ST101 *K. pneumoniae* reference genomes (Supplementary Table S1). The core genome phylogeny based on 1.9 Mb of conserved sequence and 179,342 SNPs demonstrated the position of the ST101 lineage (Figure 1) in relation to most global lineages. The retention index (RI) of the global isolate SNP alignment was 0.958, indicating little homoplasy throughout the *K. pneumoniae* core genome. The presence of recombination introduces incongruences in the phylogenetic placement of lineages with deeply branching nodes and long branches (Schierup and Hein, 2000). The PHI statistic was used to test for evidence of recombination. The *p*-value for the PHI statistic was 0.00e+00, providing evidence, but not quantification, of recombination.

A phylogeny of the ST101 lineage only, as defined by a monophyletic clade in the global phylogeny, was based on 3,992 concatenated SNPs and demonstrated little homoplasy with an RI value of 0.95, indicating that the genomes in this lineage are closely related and their evolution was minimally driven by recombination. However, the PHI statistic revealed statistically significant recombination within this dataset (*p*-value = 0.0). ClonalframeML identified 372 recombination events that spanned 138,212 bases, both monomorphic and polymorphic, across the chromosome of the ST101 lineage. Masking of these regions removed 1,882 SNPs from the ST101 core genome. The high-quality, non-recombination core genome size of the ST101 clade was 4.85 Mb.

### Population Structure in the ST101 Lineage

**FIGURE 2:**
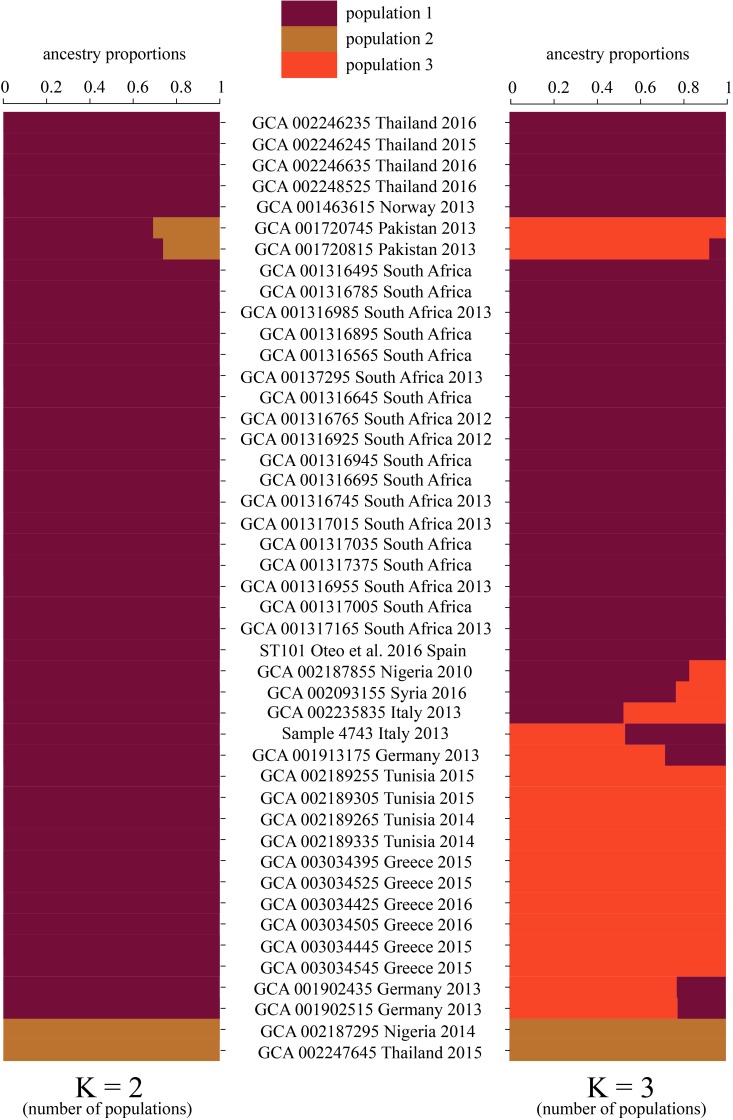
Ancestry proportions were inferred for the ST101 lineage using fastStructure v1.0 with the optimal choices of *K* (populations). Two different *K*-values (number of populations) were selected as most appropriate, *K* = 2 and *K* = 3. *K* = 2 graph represents two populations with little shared ancestry between them. Population one is represented by dark red. Population two is distinguished as brown. When running fastStructure with the number of expected populations set at three (*K* = 3), we observed limited shared ancestry.

In order to visualize the shared genomic regions within the ST101 lineage, fastStructure was applied using the ST101-only dataset that included 3,992 SNPs. We evaluated our dataset for shared ancestry using a range of K values (number of expected populations) from 2 to 10. Model complexity that maximized the marginal likelihood was 2. Model components used to explain the structure in the data was 3. When *K* = 2, fastStructure revealed two subpopulations within the ST101 lineage with limited genome sharing between the two populations; only two samples (GCA 001720745 Pakistan 2013 and GCA 001720815 Pakistan 2013) displayed genome sharing (Figure 2). For *K* = 3, fastStructure revealed limited genome sharing between two of the three of the populations; eight samples demonstrated admixture. In both models, samples GCA 002187295 (Nigeria 2014) and GCA 002247645 (Thailand 2015) make up a separate ST101 population.

### *In silico* Antimicrobial Resistance Profiling

**FIGURE 3:**
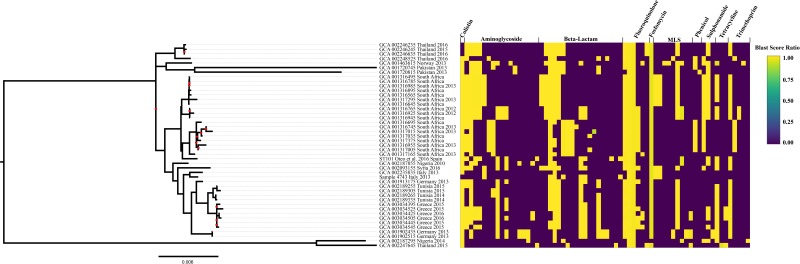
Maximum-likelihood phylogeny of the *Klebsiella pneumoniae* ST101 lineage. The tree is based on 4,077 SNPs using the K3P+ASC+G4 nucleotide substitution model and includes 45 genomes that collectively cover 85.95% of the reference genome. The PHI test found statistically significant evidence for recombination in this dataset (*P* = 0.00e+00). All samples were screened for antibiotic resistance using ResFinder. The percent identity for each resistance gene was plotted using shinyheatmap alongside the phylogenetic tree. Gene lists are in Supplementary Table S6. MLS includes genes that confer resistance to macrolides-lincosamides-streptogramin B.

As a complement to the laboratory-determined antimicrobial susceptibility profile for strain 4743 and in order to compare the ST101 lineage antimicrobial susceptibility patterns, *in silico* antimicrobial susceptibility testing was performed using the ResFinder database. Genes responsible for conferring aminoglycoside resistance, beta-lactam resistance, and fluoroquinolone resistance were conserved across all ST101 clade genomes (Figure 3 and Supplementary Table S5). Fluoroquinolone resistance genes were identified within every ST101 genome (*oqx*A, *oqx*B). Genes responsible for fosfomycin resistance were identified in 97.8% of ST101 genomes (*fosA*). Phenicol resistance genes (*cat*B4, *cat*A2, *cml*A1, *floR*) were conserved in 73% of the ST101 genomes. In total, 26% ST101 isolates carried genes responsible for macrolide, lincosamide, and streptogramin B resistance (*mphE*, *msrE*, *ereA*, *ereB*, *mphA*). Rifampicin resistant genes (*arr*-2 and *arr*-3) were present in 24% of genomes, and 71.1% of isolates carried genes responsible for sulphonamide resistance (*sul*1, *sul*2, *sul*3). Additionally, 60% of the isolates harbored genes conferring Tetracycline resistance (*tetD*, *tetX*, and *tetA*) and 86.7% carried at least one gene responsible for trimethoprim resistance (*dfrA*1, *dfrA*5, *dfrA*14, *dfrA*16, or *dfrA*27). A total of 15 different beta-lactamase genes, including ESBLs, were identified within the ST101 lineage. The most prevalent ESBL gene identified was *bla*_CTX–M–15_ (84%); *bla*_CTX–M–14_ gene was found in two ST101 genomes. The blaSHV-1 gene was identified in 87% of the ST101 samples, conferring resistance to broad spectrum beta-lactams. Sample 4743 carried the *bla*_CTX–M–15_ gene. Two genomes, sample 4743 and ST101 (Oteo et al., 2016), carried the *bla*_KPC–2_ gene (pKP048_p019, NC_014312.1). Additionally, 33.3% of the 45 genomes carried the *bla*_OXA–48_ gene. Alarmingly, 7 of 45 (15.5%) ST101 genomes contain alterations in *mgrB* gene (4 genomes harbor either a frameshift mutation or IS insertional inactivation while 3 genomes show a deletion of the *mgrB* locus) conferring colistin resistance (Esposito et al., 2018).

### Plasmid Composition

**FIGURE 4:**
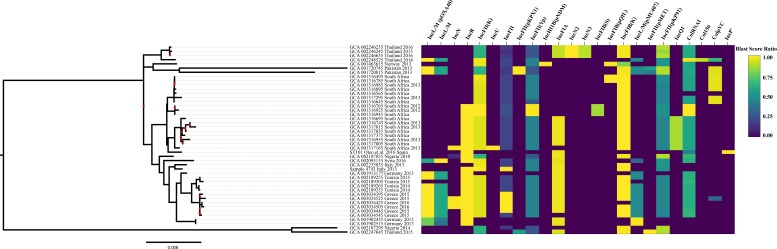
Plasmid replicon gene screening. The ST101 lineage assemblies were screened for plasmid types using PlasmidFinder. The percent identity for each plasmid typing gene was plotted using shinyheatmap alongside the maximum-likelihood phylogenetic tree.

Plasmid typing was analyzed in ST101 genomes using 122 previously characterized *K. pneumoniae* plasmids from the database PlasmidFinder. The results demonstrate the plasmid diversity across the ST101 lineage (Figure 4), although this methodology does not reveal whether the typing genes are present chromosomally or on plasmids. Plasmid replicon analysis revealed various types of plasmid incompatibility groups among the ST101 isolates, none of which were detected in all 45 ST101 samples. However, incompatibility plasmid groups FIB, FII, and R were detected in the majority of ST101 samples [IncFIB (K) in 84%, IncFII (K) in 75%, IncR in 61%, IncFIA (HI1) in 61%, and IncL/M (pOXA-48) in 29%]. The following incompatibility groups were found in 3 out of 4 plasmids identified in sample 4743 as well as one non-circular contig: IncFIB(K) (4743 plasmid unnamed 2), IncFII(K) (4743 plasmid unnamed 2), IncR (4743 plasmid unnamed 3), IncFIA (HI1) (4743 plasmid unnamed 3), IncFII (4743 plasmid unnamed 4), and ColRNAl (4743 plasmid unnamed 5). Plasmid pKP-KPC2 corresponding to 4743 plasmid unnamed 3 (111,854 nts) carried IncFII(K) and *bla*_KPC–2_ gene, plasmid pKP-CTXM-15 corresponding to 4743 plasmid unnamed 4 (67,867 nts) carried IncFII and *bla*_CTXM–15_ gene. Interestingly, the blastn alignment of plasmid pKP-KPC2 from isolate 4743 and plasmid pKP048 from ST101 *K. pneumoniae* isolate described by Oteo et al., 2016 showed that the *bla*_KPC–2_ gene is included in Tn1721 transposon structure of 14,470 nts (residues 11,810–26,279 in plasmid pKP048).

### Unique Genomic Regions

The complete gene content for 1,504 *K. pneumoniae* genomes was compared using LS-BSR. By using default values in LS-BSR, we did not identify any coding regions that were unique to the ST101 clade and absent from all others. The core genome for the ST101 lineage consisted of 4,465 coding region sequences (CDSs). The ST101 lineage accessory genome had a total of 2,247 CDSs. There were a total of 564 CDSs found only in a single genome within ST101 and the average number of unique CDSs per genome was 12.8. While these gene are unique within the ST101 lineage, all 564 CDSs were identified throughout the global *K. pneumoniae* genomes.

### Virulence and AMR Associated Genes

Known genes associated with virulence (Supplementary Table S6) from the BIGSdb-Kp database were screened against the ST101 lineage using LS-BSR and BLAT. These genes included heavy metal resistance genes, efflux pumps, colibactin and yersiniabactin genes. Additionally, we screened for the yersiniabactin-encoding mobile element ICE*Kp* as well as capsule and lipopolysaccharide serotyping. Samples within the ST101 lineage all carry the iron-scavenging siderophore yersiniabactin on the mobile genetic element ICE*Kp3* except for two samples, GCA 002187295 and GCA 002247645, which fall outside the main ST101 clade (Figure 1). The ST101 lineage was screened for a total of 489 efflux pump genes from the BIGSdb-KP database. We identified 115 conserved efflux pump genes within all ST101 samples including the virulence and antibiotic cross-resistance associated AcrAB and OqxAB efflux pumps as well as the *rarA* regulator gene (Supplementary Table S5). A total of 203 heavy metal resistance genes were screened against the ST101 lineage. The complete pcoABCDRSE operon conferring copper resistance was identified in 36 of the 44 ST101 samples while 41 of the genomes carry genes responsible for silver resistance (silCERS).

Both K typing and O typing have meaningful clinical and epidemiological significance. Capsular polysaccharide characterization (K typing) is widely used to define clinical *K. pneumoniae* isolates (Hansen et al., 2002; Vimont et al., 2008; Woodford et al., 2011). The polysaccharide capsule is often considered a virulence determinant as K-type variations have been linked to specific presence/absence of genes within the locus. Specific lipopolysaccharide antigens (O typing) also contribute to *K. pneumoniae* pathogenicity. In an effort to better understand the pathogenicity of the ST101 clone, we performed both K and O typing using the program Kaptive Web. Limited capsular diversity was observed with 43 samples having the KL17 loci and 2 samples with the KL106 loci. O loci typing revealed the majority of the ST101 lineage as O1V1. Interestingly, the O1 antigen has been previously described as a major contributor to the virulence of pyogenic liver abscess causing *K. pneumoniae*. Three ST101 samples (GCA 001316955, GCA 002189265, GCA 001316985) were typed as O1/O2v1. O1/O2 signifies that either gene *wbbY* or *wbbZ* could not be found within the sample (Wick et al., 2018).

### QRDR Mutations

All of the ST101 genomes but two (GCA_002187295 and GCA_002247645) showed amino acid substitutions in codon 83 (Ser83Tyr) and codon 87 (Asp87Gly, Asp87Asn, Asp87Ala) of the *gyrA* gene (Supplementary Table S7). Additionally, a Ser80ILe substitution in the *parC* gene was detected in all ST101 genomes with the exception of two samples (GCA_002187295 and GCA_002247645). Amino acid substitutions within QRDR of the *parE* or the *gyrB* genes were not observed in any of the ST101 genomes.

### Beast Analysis

The ST101-only Bayesian dataset consisted of 35 genomes with reported collection dates from NCBI and the newly sequenced sample 4743. This dataset contained 3,977 SNPs, with 1,889 SNP positions falling within genomic regions determined by ClonalframeML as recombining and subsequently removed from this analysis. The root-to-tip regression analysis identified weak clocklike behavior (*R*^2^ = 0.025) within the ST101 lineage with 2.5% of the diversity explained by time. However, we believe we are capturing little of the true temporal signal due to our narrow sampling dates (2010–2016) and small sample size (*n* = 36). Given the positive regression slope and the detection of a clocklike signal, we determined molecular clock analysis was appropriate and reliable for SNP accumulation rate estimation (Duchêne et al., 2016). MEGA7 identified the best fitting nucleotide substitution model as the GTR model and was applied in the BEAST analysis. In an effort to determine if the observed *R*^2^ value was better than random chance, a 10,000-date permutation testing on the recombination removed ST101-only dataset. This testing produced a *P* value of 0.339, indicating that our observed *R*^2^ value was not statistically different than random chance.

**FIGURE 5:**
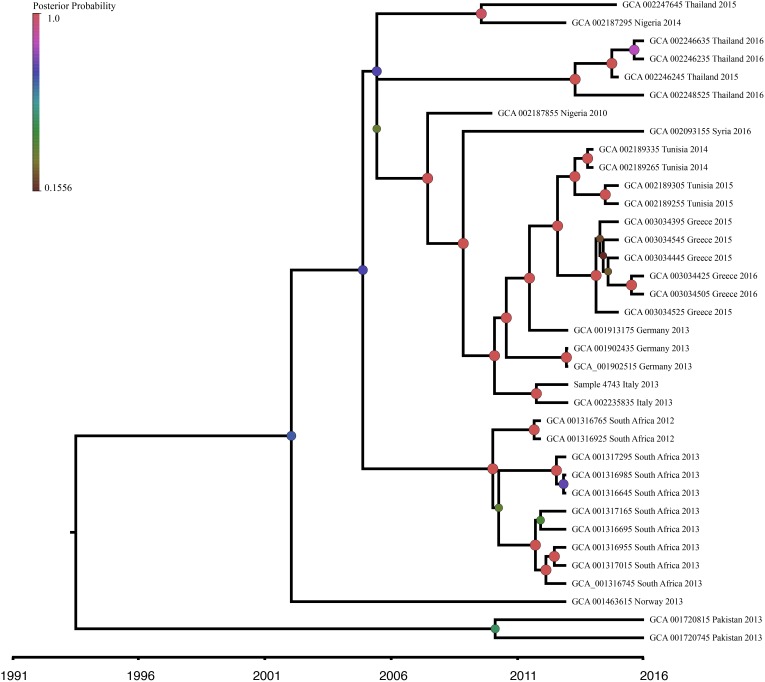
Bayesian phylogenetic analysis of *K. pneumoniae* ST101 isolates. Beast v1.8.4 was used to produce a calibrated maximum clade credibility (MCC) phylogeny. The analysis was performed on WGS data from 36 *K. pneumoniae* ST101 genomes. Posterior probabilities are indicated by node size and color. The timeline represents years before the present.

In order to investigate the evolution of *K. pneumoniae* ST101, BEAST analysis was performed using the recombination-free, high quality SNP dataset previously described. The constant population demographic model with a relaxed molecular clock was selected as the most appropriate model and clock combination to describe ST101 evolution. Molecular clock calibration estimated the evolutionary rate for the ST101 lineage as 2.8527 × 10^−6^ substitutions per site per year (95% highest posterior density [HPD], 1.0762 × 10^−6^ to 4.7396 × 10^−6^). The mean time to most recent common ancestor (TMRCA) was estimated at 26.6 years ago (95% HPD, 8.35 to 51.24 years ago) from the time of the last sample date, which was 2016 (Figure 5). This dataset has narrow sampling dates with a limited number of samples and it is likely we are capturing very little of the temporal signal. Given a wider sampling time and additional samples, we would likely narrow our MRCA estimate.

## Discussion

The focus of this study was to perform a comprehensive genomics analysis of the ST101 lineage to better understand the genomic content of this newly emerging, extensively resistant lineage. Previous studies have identified this sequence type as high risk for furthering the spread of carbapenem resistance (Del Franco et al., 2015; Oteo et al., 2016). Here we demonstrate that ST101 is a distinct lineage in which one sample in this study, 4743, acquired the KPC-2 enzyme. Additionally, a recent study identified 13 ST101 samples carrying the KPC-2 gene (WGS data not yet available) (Del Franco et al., 2015; Oteo et al., 2016). The inconsistent carriage of KPC-2 within the ST101 clone suggest the KPC enzyme was acquired through horizontal transfer of plasmids after the divergence of the ST101 lineage rather than vertical gene transfer among the entire lineage. In further support of this, our data showed that ST101 *K. pneumoniae* isolate 4743 and ST101 isolate described by Oteo et al., 2016 both carry plasmids containing the *bla*_KPC–2_ gene into Tn1721 transposon.

We found multiple genomic features that may provide this clone with an advantage for adaptation within a hospital environment as well as a human host. Multiple putative virulence factors were identified throughout the ST101 lineage including the siderophore genes *irp1* and *irp2*, the yersiniabactin receptor gene *fyuA*, the ICE*Kp*-3 element that carries the yersiniabactin siderophore cluster (ybtAEPQSTUX), the mannose-resistant *Klebsiella*-like (type III) fimbriae cluster (mrkABCDFHIJ), and the ferric uptake system (kfuABC). The *irp* and *irp2* genes encode iron-repressible high molecular weight proteins that are involved with yersiniabactin production (Schubert et al., 1998); these were identified in all but two samples within the ST101 lineage. The *fyuA* (ferric yersiniabactin uptake) gene is also involved with the iron-acquiring yersiniabactin system by coding for yersiniabactin receptors (Schubert et al., 1998). In *E. coli*, the *fyuA* gene is required for biofilm formation in urinary tracts and is important for disease establishment in iron-poor environments such as the urinary tract (Hancock et al., 2008). The ST101 lineage also carry the yersiniabactin siderophore cluster on an ICE, which has been identified as a frequent virulence factor in *K. pneumoniae* (Schubert et al., 2004; Holt et al., 2015; Lam et al., 2018). The ICEKp element increases the ST101 clone’s ability to cause disease by coding for an iron scavenging system; this element also allows this clone to further spread this important virulence factor because it is located on an integrative conjugative element (Lam et al., 2018). The mannose-resistant *Klebsiella*-like (type III) fimbriae cluster (mrkABCDFHIJ) is considered a virulence factor and has been previously described in several species of *Enterobacteriaceae* as contributors to mucous adherence, tissue colonization, and biofilm formation (Monroy et al., 2005; El Fertas-Aissani et al., 2013). Finally, the ferric uptake system (kfuABC) is also associated with increased virulence (Ma et al., 2005). In pyogenic liver abscess causing *K. pneumoniae*, the kfu system is described as usually more prevalent in invasive tissue strains, however, the presence of multiple iron-acquisition systems suggests the ST101 lineage is capable of acquiring and using iron from diverse sources similar to pyogenic liver causing strains (Ma et al., 2005; Hsieh et al., 2008). Previous research suggests iron acquisition gene diversity allows for more capable environmental iron acquisition (Lee et al., 2012). This increased efficiency of iron uptake has been hypothesized to result in increased capsule production, which is an important phenotype of hypervirulence (Russo et al., 2011; Chaturvedi et al., 2012; Shon et al., 2013; Moradigaravand et al., 2017).

Hyper-virulent and drug-resistant *K. pneumoniae* populations remain mostly non-overlapping, although combinations of these groups have been previously reported (Bialek-Davenet et al., 2014a; Gu et al., 2018). Here we demonstrate the presence of several known virulence factors within the ST101 lineage as well as highlight the extensive drug resistance gene repertoire this clone harbors (colistin, beta-lactams, aminoglycosides, fluoroquinolones, and fosfomycin). The ST101 lineage is an example of a dual-risk clone combining the genetic profiles of hypervirulent *K. pneumoniae* as well as extensive drug resistant strains. Alarmingly, the ST101 resistome is similar to the antibiotic resistant profile of the global ST258 lineage. The majority of both ST101 and ST258 clones possess genes that are responsible for resistance to aminoglycosides [aac(6′), aph(6′), aadA genes], third generation-cephalosporins and aztreonam (*bla*CTXM), fosfomycin (*fosA*), and fluoroquinolones [aac(6′)-Ib-cr, *oqxA*, *oqxB*, *qnrB*, *qnrS* genes]. Both lineages confer resistance to colistin due to inactivation of the *mgrB* gene and to carbapenems due to the acquisition of the *bla*KPC-2 gene. Both lineages carry the mannose-resistant *Klebsiella*-like (type III) fimbriae cluster (mrkABCDFHIJ), and the ICE*Kp* containing the yersiniabactin siderophore cluster (ybtAEPQSTUX). While drug-resistance has been shown to be associated with a large loss of fitness, researchers have demonstrated the presence of three amino acid substitutions within QRDR of *parC* and *gyrA* genes that result in no loss of vitality in multiple bacterial species, including *K. pneumoniae* (Marcusson et al., 2009; Toth et al., 2014). In addition to extensive drug resistance, genomes from the ST101 lineage harbor these three amino acid substitutions within the *gyrA* and *parC* genes. *K. pneumoniae* minor sequence types that are fluoroquinolone resistant were reported to lack these mutations entirely, carry a “non-serine” mutation, or only one of the three mutations (Fuzi et al., 2017). However, major clones, specifically ST258, were all shown to carry the *gyrA* Ser83Ile and *parC* Ser80Ile double mutations (Bowers et al., 2015). Given the global success of ST258 and similar virulence, antibiotic resistance, and QRDR mutation profiles compared to the ST101 lineage, this newly emerging clone has the potential to become a global epidemic dual-risk clone and major public health threat.

## Data Availability

The datasets generated for this study can be found in NCBI, PRJNA477005.

## Ethics Statement

The study was approved by the ethics committee of the Aosta Regional Hospital (protocol number 836/2015). All microbiological samples were taken as part of standard care procedures. Patients included in the study were anonymized, no written informed consent was acquired because of the retrospective nature of the study.

## Author Contributions

CR analyzed the data and wrote the manuscript. AV and EE performed laboratory analyses. RZ designed the study and helped to write the manuscript. JS designed the study, analyzed the data, and helped to write the manuscript.

## Conflict of Interest Statement

The authors declare that the research was conducted in the absence of any commercial or financial relationships that could be construed as a potential conflict of interest.

## Footnotes

1 https://gist.github.com/jasonsahl/2eedc0ea93f90097890879e56b0c3fa3

2 http://github.com/kelwyres/Kaptive-Web

3 http://tree.bio.ed.ac.uk/software/figtree/
